# Emergence of ST11-KL64 carbapenem-resistant hypervirulent *Klebsiella Pneumoniae* isolates harboring *bla*_KPC−2_ and *iucA* from a tertiary teaching hospital in Western China

**DOI:** 10.1186/s12879-025-11241-6

**Published:** 2025-07-01

**Authors:** Haojun Chen, Tingting Li, Xiaoxue Huang, Qiurong He

**Affiliations:** 1 Department of Laboratory Medicine, West China School of Public Health and West China Fourth Hospital, Sichuan University, Chengdu, 610041 China; 2 Department of Laboratory Medicine, West China Lecheng Hospital, Sichuan University, Qionghai, 571421 China

**Keywords:** Hypervirulent, Carbapenem-resistant *Klebsiella pneumonia*, Multilocus sequence typing, Risk factors

## Abstract

**Background:**

Carbapenem-resistant hypervirulent *Klebsiella pneumoniae* (CR-HvKP) poses a critical global health threat. However, molecular epidemiological data on CR-HvKP in Western China remain scarce. This study aimed to characterize the clinical profiles, molecular features, and risk factors of CR-HvKP isolates in Western China.

**Methods:**

Sixty-eight carbapenem-resistant *Klebsiella pneumoniae* (CRKP) clinical isolates were collected from January to December in 2024. Clinical characterization included antimicrobial susceptibility profiling and hypermucoviscosity assessment via string test. Detection of carbapenemases using inhibitor enhancement test. Molecular characteristics of CRKP included serotype, carbapenemases, virulence-associated factors, and multilocus sequence typing (MLST) performed by using the PCR method. CR-HvKP was defined as the presence of any one of *rmpA*, *rmpA2*, *iroB*, *iucA*, and *peg-344*. Risk factors were initially evaluated using univariate logistic regression analysis, with significant variables subsequently incorporated into a multivariate regression model. A *p*-value < 0.05 was considered statistically significant.

**Results:**

Among 68 CRKP isolates, 36 were identified as CR-HvKP, all harboring the *iucA* gene (100%, 36/36). However, only 22.2% (8/36) of string test results correlated with virulence gene presence. All CRKP strains exhibited high resistance to most antibiotics, with comparatively lower resistance rates observed for tigecycline (0%) and polymyxin B (14.7%). Carbapenemase production was the predominant resistance mechanism, with 61.8% (42/68) carrying *bla*_KPC−2_. Serotyping and MLST revealed that ST11-KL64 CR-HvKP being predominant. A novel *wzi752* allele was identified, encoding amino acid sequences homologous to serotype KL47. Univariate analysis demonstrated significantly higher ICU admission rates (*p* = 0.018) and carbapenem exposure (*p* = 0.023) in CR-HvKP patients with infections. Multivariate analysis highlighted borderline significance for ICU admission (OR = 2.939, *p* = 0.056) as a potential risk factor.

**Conclusions:**

The ST11-KL64 CR-HvKP clone harboring *bla*_KPC−2_ and *iucA* has emerged as a dominant pathogen of hospital infections in Western China, posing dual threats of resistance and virulence. Enhanced molecular surveillance and infection control strategies are urgently needed to mitigate its spread.

**Supplementary Information:**

The online version contains supplementary material available at 10.1186/s12879-025-11241-6.

## Background

*Klebsiella pneumoniae* (*K. pneumoniae*) is a prominent nosocomial pathogen. Over recent decades, antimicrobial-resistant strains of *K. pneumoniae*, particularly carbapenem-resistant *K. pneumoniae* (CRKP), have emerged and disseminated on a global hospital [1]. In contrast to CRKP, hypervirulent *K. pneumoniae* (HvKP) is predominantly a community-acquired pathogen characterized by its high invasiveness. HvKP frequently causes severe infections, including meningitis, hepatopancreatic abscesses, endophthalmitis, and soft tissue infections [2]. Importantly, the World Health Organization (WHO) has recently announced a ‘superbug’ variant, carbapenem-resistant hypervirulent *K. pneumoniae* (CR-HvKP), which combines two distinct evolutionary traits, namely multidrug resistance and hypervirulence [3]. This novel pathogen poses a significant global public health threat due to its association with markedly elevated drug resistance and mortality rates [4].

CRKP can be efficiently identified using Matrix-Assisted Laser Desorption/Ionization-Time of Flight Mass Spectrometry (MALDI-TOF MS) or VITEK automated identification and antimicrobial susceptibility testing systems. Historically, HvKP has been identified based on hypermucoviscous phenotypic characteristics, such as a positive string test with a length exceeding 5 mm [5]. However, this phenotypic approach has limitations and may result in underdiagnosis of HvKP strains, as studies have demonstrated a poor correlation between the hypermucoviscous phenotype and the actual virulence potential of *K. pneumoniae* [6, 7]. Research by Russo et al. [8] highlighted that identifying virulence factors encoded on the virulence plasmid provides superior diagnostic accuracy, achieving over 95% reliability in identifying HvKP strains. This molecular approach involves the detection of multiple key virulence genes, including *rmpA* and *rmpA2* (associated with the hypermucoviscous phenotype) and *iroB*, *iucA*, and *peg-344* (linked to enhanced pathogenicity) [9].

CR-HvKP represents a significant evolutionary milestone in the pathogen’s adaptation and pathogenicity, likely driven by the acquisition of exogenous virulence plasmids by CRKP strains or the integration of resistance-associated plasmids by HvKP strains [10]. First identified in China by Zhang et al. [11] in 2015, CR-HvKP has since demonstrated an epidemic trajectory, primarily in healthcare settings across diverse regions [12–14]. Recent investigations have demonstrated its potential for widespread dissemination across hospitals, environmental reservoirs and fresh oysters [15, 16]. Moreover, the vast geographical expanse of Western China, characterized by challenging topographical features and limited healthcare infrastructure, poses significant public health challenges for the local population.

Continuous surveillance of CR-HvKP is essential for the prevention and management of associated infections. However, molecular epidemiological data on CR-HvKP in Western China remain scarce. This study aims to elucidate the clinical characteristics, risk factors, and molecular profiles of CR-HvKP isolates in a tertiary teaching hospital in Western China, addressing the current paucity of epidemiological data from this region.

## Materials and methods

### Source, identification, and clinical data collection

All *K. pneumoniae* isolates were collected from January to December in 2024 at the Fourth Hospital of West China, Sichuan University, operating approximately 1,000 beds. The isolates from clinical residual specimens were identified as *K. pneumoniae* using MALDI-TOF MS (BioMérieux, France) or the VITEK^®^ 2 COMPACT system (BioMérieux, France). CRKP was defined as resistance to at least one carbapenem antibiotic, including imipenem or meropenem. CR-HvKP was further identified as CRKP strains harboring at least one virulence-associated gene, such as *rmpA*, *rmpA2*, *iroB*, *iucA*, or *peg-344*. All CRKP isolates were subsequently categorized into CR-HvKP and CR-non-HvKP groups. Duplicate isolates obtained from the same specimen type of the same patient were excluded to ensure data integrity. Clinical data were extracted from the Hospital Information System and Laboratory Information System. The dataset included demographic characteristics, hospital department, infection site, underlying conditions, invasive procedures, antibiotic exposure, and clinical outcomes.

### Antimicrobial susceptibility testing

Antimicrobial susceptibility testing for all CRKP isolates was conducted using the VITEK^®^ 2 COMPACT system. The tested antimicrobials included ampicillin, ampicillin/sulbactam, piperacillin/tazobactam, cefalotin, ceftazidime, cefepime, cefoperazone/sulbactam, imipenem, meropenem, aztreonam, ciprofloxacin, levofloxacin, amikacin, tobramycin, trimethoprim/sulfamethoxazole, tetracycline, tigecycline, and polymyxin B. The results were interpreted in accordance with the Clinical and Laboratory Standards Institute (CLSI) M100-ED33 breakpoint criteria [17]. For isolates demonstrating intermediate or resistance to tigecycline and polymyxin B, minimum inhibitory concentrations (MICs) were confirmed via the broth microdilution method. The interpretation of tigecycline susceptibility results was performed according to the U.S. Food and Drug Administration (FDA) breakpoint criteria [18]. Quality control measures utilized *Pseudomonas aeruginosa* ATCC 27853 and *Escherichia coli* ATCC 25922 strains, purchased from BeNa Culture Collection (BNCC, Beijing, China).

### Carbapenemase inhibitor enhancement test

Carbapenemase production was characterized using the disk diffusion method combined with inhibitor enhancement testing. Bacterial suspensions were adjusted to a turbidity equivalent to 0.5 McFarland standard and uniformly inoculated onto Mueller-Hinton agar plates using sterile cotton swabs. Four disks containing either imipenem or meropenem were applied to each plate as follows: One disk without any inhibitor (control), one disk supplemented with 10 µL of 50 mg/mL 3-aminophenylboronic acid, one disk supplemented with 10 µL of 0.1 mol/L EDTA, and one disk with both APB and EDTA. Plates were incubated at 37℃ for 18 h under ambient air. Inhibition zone diameters were measured using a vernier caliper. An increase in zone diameter of ≥ 5 mm compared to the control was interpreted as positive for carbapenemase activity: enhancement with APB suggests class A β-lactamase production, while enhancement with EDTA indicates the presence of metallo-β-lactamases. ATCC 25922 strains and sequence-verified *K. pneumoniae* strains were used as quality control strains.

### String test

The string test was initially developed to identify the hypermucoviscous phenotype of *K. pneumoniae* [19], which assists clinicians and microbiologists in recognizing virulent isolates. In this test, bacterial colonies were collected from culture plates using an inoculation loop and gently lifted upward. A positive result was defined by the formation of a viscous string exceeding 5 mm in length, indicative of the isolate’s hypermucoviscous phenotype.

### DNA Preparation

CRKP isolates stored at −80°C were revived by transferring a single bead containing the bacterial suspension onto Columbia blood agar plates, followed by incubation at 37 °C for approximately 18 h. The next day, bacterial suspensions were standardized to a turbidity equivalent to a 1.0 McFarland standard. Subsequently, 1 mL of each suspension was transferred to a 1.5 mL microcentrifuge tube and centrifuged at 12,000 rpm for 2 min, after which the supernatant was discarded. Genomic DNA was extracted using the MiniBEST Bacteria Genomic DNA Extraction Kit Ver. 3.0 (TaKaRa, Dalian, China) in accordance with the manufacturer’s protocol. Briefly, bacterial cells were lysed with Buffer GL, Proteinase K, and RNase A at 56 °C for 10 min. After lysis, Buffer GB and absolute ethanol were added to the lysate, and the mixture was transferred to a spin column to facilitate DNA binding. Impurities were removed through multiple washing steps, and the genomic DNA was eluted and collected. The extracted DNA was stored at −20°C for further use.

### Virulence genes detection

Primers targeting five virulence-associated genes of *K. pneumoniae*—*rmpA*, *rmpA2*, *iroB*, *iucA*, and *peg-344*—were designed based on previously published study [8, 20] (primer provided in Table S1). PCR amplification was carried out using extracted bacterial DNA, primers (BGI, Chongqing, China), and Premix Taq™ (TaKaRa, Dalian, China) following the manufacturer’s instructions. The amplification products were analyzed semi-quantitatively by electrophoresis on a 2% agarose gel, with the DL1000 DNA Marker (TaKaRa, Dalian, China) serving as a molecular weight reference.

### Serotyping

Capsular polysaccharide serotyping was conducted through PCR amplification of the *wzi* gene [21], with primers detailed in Table S1. Positive amplicons, identified by electrophoresis on a 2% agarose gel, were sequenced by BGI. The resulting sequences were compared against the INSTITUT PASTEUR database (https://bigsdb.pasteur.fr/klebsiella/) to determine serotypes, namely KL types. Novel alleles differing from those cataloged in the database were submitted to the database curator for the assignment of new locus numbers.

### Carbapenemase genes detection

All CRKP isolates were screened for five common carbapenemase-encoding genes—*bla*_KPC_, *bla*_NDM_, *bla*_OXA−48_, *bla*_VIM_, and *bla*_IMP_—based on previous protocols [20] (Table S1). Positive amplicons, identified by electrophoresis on a 2% agarose gel, were sequenced by BGI. The resulting sequences were analyzed using the NCBI BLAST database (https://blast.ncbi.nlm.nih.gov/Blast.cgi) to identify specific carbapenemase variants. Novel sequences were submitted for registration to the NCBI database through BankIt (https://www.ncbi.nlm.nih.gov/WebSub/).

### Multilocus sequence typing

Multilocus sequence typing (MLST) was conducted using amplification and sequencing primers obtained from the *K. pneumoniae* MLST database (https://bigsdb.pasteur.fr/klebsiella/primers-used/). Seven housekeeping genes (*rpoB*, *gapA*, *mdh*, *pgi*, *phoE*, *infB*, and *tonB*) were amplified via PCR, purified, and sequenced (Table S1). Multiple sequence alignment was performed using MEGA X software [22], and the aligned sequences were queried against the MLST database to assign allelic profiles and sequence types (STs). Novel alleles or STs not present in the database were submitted to the database curator for the assignment of new designations. A phylogenetic tree based on concatenated core genetic sequences was constructed using the Maximum Likelihood method with 1,000 bootstrap replicates and the Hasegawa-Kishino-Yano substitution model incorporating gamma distributed with invariant sites in MEGA X software. The tree was subsequently visualized, annotated, and enhanced using Interactive Tree Of Life (iTOL) v6 [23]. To analyze the relationships between molecular characteristics, the goeBURST algorithm was applied using PHYLOViZ 2.0 software [24].

### Statistical analysis

Statistical analyses were performed using SPSS Statistics version 27.0 (IBM, NY, USA). Continuous variables were presented as median and interquartile range, while categorical variables were expressed as frequencies and percentages. The Mann-Whitney U test was used to compare continuous variables between groups. Categorical variables were compared using either Pearson’s chi-square test or Fisher’s exact test, as appropriate. A two-sided *p*-value < 0.05 was considered statistically significant. Potential risk factors were initially identified through univariate logistic regression analysis. Variables that achieved statistical significance in the univariate analysis were subsequently included in a multivariate logistic regression model to identify independent risk factors.

Matrix assistedlaser desorption ionization time of flight mass spectrometry; Antimicrobial susceptibility test; Carbapenem-resistant *K. pneumoniae*; Carbapenem-resistant hypervirulent *K. pneumoniae*; Multilocus sequence type.

## Results

### Clinical characteristics and virulence-associated factors

**Fig. 1:**
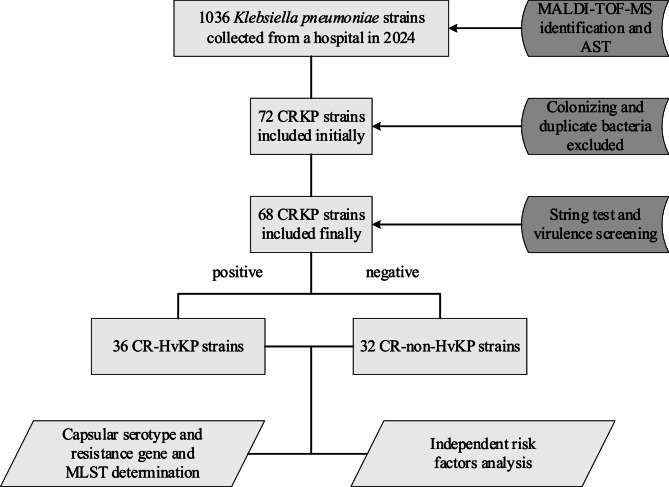
Flowchart of inclusion and exclusion criteria for CR-HvKP strains. MALDI-TOF MS, Matrix assisted laser desorption ionization time of flight mass spectrometry; AST, Antimicrobial susceptibility test; CRKP, Carbapenem-resistant *K. pneumoniae*; CR-HvKP, Carbapenem-resistant hypervirulent *K. pneumoniae*; MLST, Multilocus sequence type

**Fig. 2:**
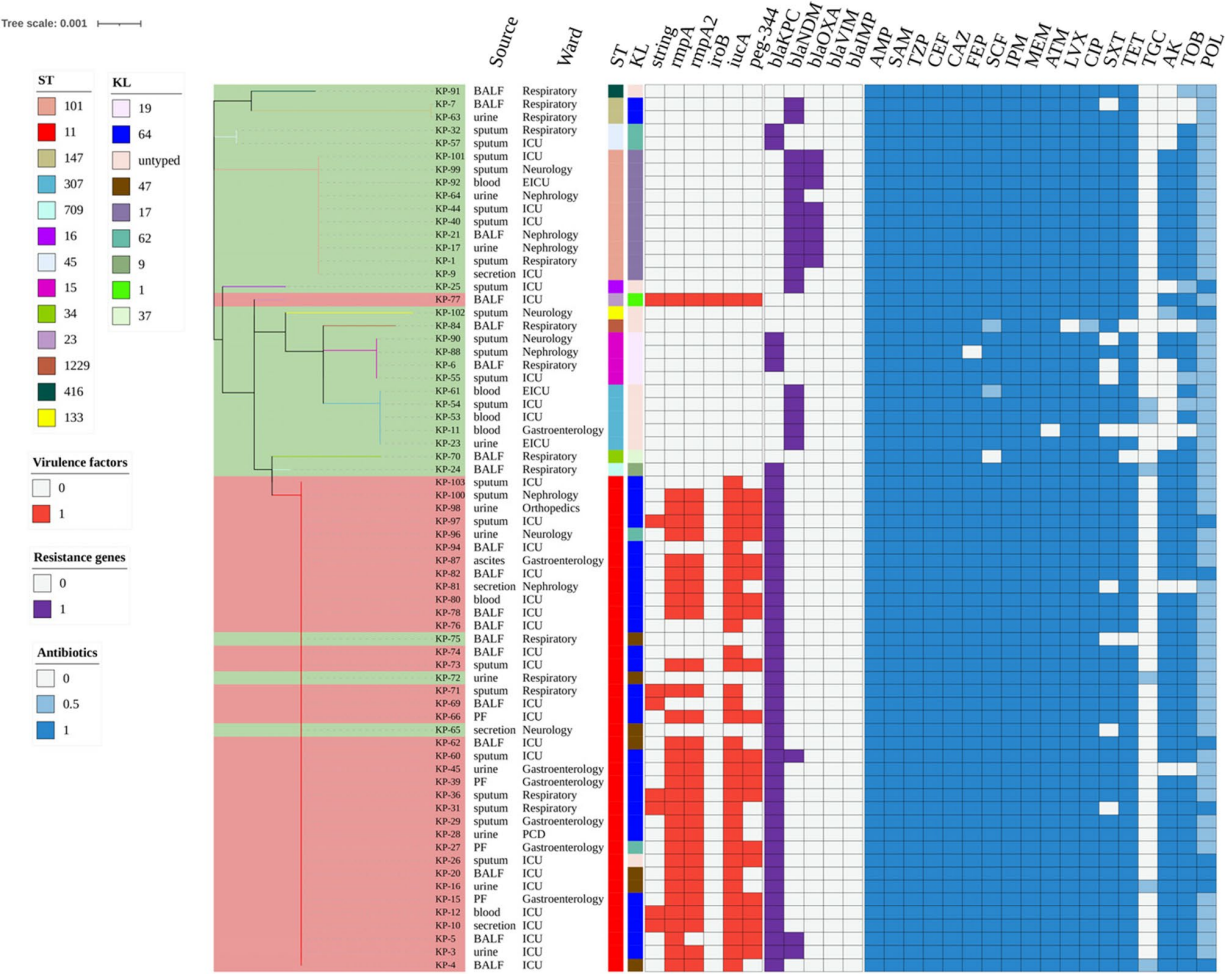
Phylogenetic tree based on concatenated core genetic sequences of 68 CRKP strains. Trees are color-coded as follows: light red for CR-HvKP strains and light green for CR-non-HvKP strains. STs and KLs are color-coded. Virulence factors are indicated in red, and resistance genes in purple. Antibiotic resistance and intermediate susceptibility are shown in blue and light blue, respectively. ST, Sequence type; KL, K locus; AMP, ampicillin; ASM, ampicillin/sulbactam; TZP, piperacillin/tazobactam; CEF, cefalotin; CAZ, ceftazidime; FEP, cefepime; SCF, cefoperazone/sulbactam; IPM, imipenem; MEM, meropenem; ATM, aztreonam; CIP, ciprofloxacin; LVX, levofloxacin; AK, amikacin; TOB, tobramycin; SXT, trimethoprim/sulfamethoxazole; TET, tetracycline; TGC, tigecycline; POL, polymyxin B

The strains screening workflow is depicted in Fig. 1. A total of 68 non- repetitive clinical CRKP isolates were collected from 62 patients at West China Fourth Hospital from January to December in 2024. Multiple isolates were obtained from different specimen types in the same patients. The patient cohort comprised predominantly males (38/62, 61.3%), with a median age of 62 years. The intensive care unit (ICU) accounted for the largest proportion of cases (26/62, 41.9%). Among the 68 CRKP isolates, respiratory specimens were the primary source, including sputum (23/68, 33.8%) and bronchoalveolar lavage fluid (18/68, 26.5%), followed by urine (12/68, 17.6%), blood (6/68, 8.8%), puncture fluid (5/68, 7.4%), and secretions (4/68, 5.9%). Based on the presence of five specific virulence genes, the isolates were categorized into CR-HvKP (*n* = 36) and CR-non-HvKP (*n* = 32) strains (Fig. 2).

The clinical characteristics of CRKP-infected patients are summarized in Table 1. While no significant differences were observed in infection type, underlying conditions, invasive procedures, or clinical outcomes between groups (*p* > 0.05), notably higher rates of carbapenem use and ICU admission were found in the CR-HvKP group compared to the CR-non-HvKP group (68.8% vs. 40.0%, *p* = 0.023; 56.3% vs. 26.7%, *p* = 0.018, respectively).

**Table 1:** Clinical characteristics of 62 patients with CRKP infection

Characteristic	CR-HvKP (*n*, %) (*n* = 32)	CR-non-HvKP (*n*, %) (*n* = 30)	*p*-value
*Basic demographics*			
Age^a^	62 (45, 73)	64 (50, 78)	0.294
Male	20 (62.5)	18 (60.0)	0.840
*Inpatient departments*			
ICU	18 (56.3)	8 (26.7)	**0.018**^b^
Respiratory	3 (9.4)	10 (33.3)	**0.029**^b^
Gastroenterology	6 (18.8)	1 (3.3)	0.105
Neurology	1 (3.1)	4 (13.3)	0.189
Nephrology	2 (6.3)	4 (13.3)	0.418
*Infection types*			
Pneumonia	21 (65.6)	23 (76.7)	0.338
Bacteremia	1 (3.1)	3 (10.0)	0.346
Urinary infection	4 (12.5)	2 (6.7)	0.672
*Underlying diseases*			
Diabetes	8 (25.0)	10 (33.3)	0.470
Hypertension	16 (50.0)	15 (50.0)	1.000
Cardiovascular disease	8 (25.0)	10 (33.3)	0.470
Cerebrovascular disease	5 (15.6)	10 (33.3)	0.176
Pulmonary disease	13 (40.6)	10 (33.3)	0.553
Liver disease	7 (21.9)	6 (20.0)	0.856
Kidney disease	8 (25.0)	6 (20.0)	0.638
Malignant tumor	4 (12.5)	1 (3.3)	0.355
*Invasive procedures*			
Mechanical ventilation	21 (65.6)	21 (70.0)	0.713
Urinary catheter	22 (68.8)	22 (73.3)	0.691
Central venous catheter	13 (40.6)	12 (40.0)	0.960
Gastric tube	13 (40.6)	19 (63.3)	0.074
*Prior antibiotic exposure*			
Cephalosporins	12 (37.5)	8 (26.7)	0.362
β-lactam-β-lactamase inhibitors	22 (68.8)	25 (83.3)	0.180
Carbapenems	22 (68.8)	12 (40.0)	**0.023**^b^
Aminoglycosides	3 (9.4)	7 (23.3)	0.176
Fluoroquinolones	8 (25.0)	12 (40.0)	0.207
Tigecycline	14 (43.8)	12 (40.0)	0.765
Polymyxin B	6 (18.8)	1 (3.3)	0.105
*Clinical outcomes*			
Improvement	18 (56.3)	17 (56.7)	0.974
Death	5 (15.6)	4 (13.3)	1.000
Transferring	9 (28.1)	9 (30.0)	0.871

Data are presented as frequencies and percentages, unless otherwise stated

^a^ Age is presented as median and interquartile range

^b^ Bold indicates *p*-value < 0.05

*ICU* intensive care unit

The distribution of virulence genes is shown in Fig. 2. Among the virulence genes of CR-HvKP strains, *iucA* was most prevalent (36/36, 100.0%), followed by *rmpA* (31/36, 86.1%), *rmpA2* (30/36, 83.3%), *peg*-*344* (22/36, 61.1%), and *iroB* (1/36, 2.8%). A single isolate harbored all five virulence genes (*rmpA* + *rmpA2* + *iroB* + *iucA* + *peg*-*344*). The predominant virulence gene pattern was *rmpA* + *rmpA2* + *iucA* + *peg*-*344* (20/36, 55.6%), followed by *rmpA* + *rmpA2* + *iucA* (9/36, 25.0%). Interestingly, the string test identified significantly fewer hypervirulent strains (8/68, 11.8%) compared to molecular detection of CR-HvKP strains (36/68, 52.9%). Among the 8 hypermucoviscous isolates, 75% (6/8) were isolated from respiratory specimens. Comparative analysis with the 60 non-hypermucoviscous isolates revealed no statistically significant association between hypermucoviscosity and infection site (*p* > 0.05). Despite the numerical predominance of respiratory specimens in hypermucoviscous strains, these findings suggest that the hypermucoviscous phenotype does not exhibit a preferential tropism for specific infection site in our CRKP cohort.

Capsular typing identified eight distinct serotypes among the 68 CRKP isolates, including a novel serotype locus (*wzi 752*) present in four isolates. Although specific *wzi* types were detected, such as *wzi 173* (5/68, 7.4%), we were still unable to identify the specific serotypes. The complete KL typing results are summarized in Table S4. Analysis of capsular types revealed KL64 as the predominant type (30/68, 44.1%), followed by KL17 (10/68, 14.7%). Distribution analysis between groups showed that KL64 was significantly more prevalent in CR-HvKP isolates compared to CR-non-HvKP isolates (77.8% vs. 6.3%, *p* < 0.001). Conversely, KL17 and KL19 were exclusively detected in the CR-non-HvKP group (31.3% vs. 0%, *p* < 0.001; 12.5% vs. 0%, *p* = 0.044, respectively).

### Antimicrobial susceptibility results, carbapenemase production, and carbapenemase-associated genes

Antimicrobial susceptibility testing was performed on all CRKP clinical isolates against a panel of eighteen antibiotics (Table S2). The isolates demonstrated extensive resistance to multiple antibiotic classes, including cephalosporins, β-lactam/β-lactamase inhibitor combinations, carbapenems, fluoroquinolones, and aminoglycosides. Among all tested antimicrobials, only tigecycline and polymyxin B retained appreciable in vitro activity, with resistance rates of 0% and 14.7% (10/68), respectively. Comparative analysis revealed that CR-HvKP isolates demonstrated significantly higher resistance rates to trimethoprim-sulfamethoxazole (94.4% vs. 78.1%, *p* = 0.047), amikacin (94.4% vs. 53.1%, *p* < 0.001), and tobramycin (94.4% vs. 75.0%, *p* = 0.046) than CR-non-HvKP isolates. However, there was no statistical difference in polymyxin B (22.2% vs. 6.3%, *p* = 0.089).

Carbapenemase phenotypes and corresponding genotypes are summarized in Table 2. Overall, 95.6% (65/68) of CRKP isolates exhibited concordance between phenotype and genotype. Notably, two isolates harbored *bla*_KPC−14_, a variant of *bla*_KPC−2_, which may compromise enzymatic activity and account for the non-carbapenemase-producing phenotype. Additionally, one isolate exhibited a class A β-lactamase phenotype without a detectable genotype, suggesting the possible presence of a non-*bla*_KPC_-encoded enzyme. Comprehensive data are provided in Table S3.

**Table 2:** Detection of carbapenemases on all 68 CRKP strains by disk diffusion using 4 different Imipenem disks (*n*, %)

CRKP strains	Imipenem + EDTA (DD ≥ 5 mm)	Imipenem + APB (DD ≥ 5 mm)	Imipenem + EDTA + APB (DD ≥ 5 mm)
*bla*_KPC_ (*n* = 41)	0	39 (95.1)	0
*bla*_NDM_ (*n* = 18)	18 (100)	0	0
*bla*_KPC+NDM_ (*n* = 3)	0	0	3 (100)

*CRKP* carbapenem-resistant *K. pneumoniae*, *DD* disk diffusion zone diameter, *APB* aminophenylboronic acid

The distribution of carbapenemase-encoding genes among the isolates is summarized in Fig. 2. Among the CRKP strains, *bla*_KPC_ was the predominant carbapenemase gene (44/68, 64.7%), with *bla*_KPC−2_ being the most prevalent variant (42/44, 95.5%). This was followed by *bla*_NDM_ (21/68, 30.9%) and *bla*_OXA−48_ (8/68, 11.8%). Notably, neither *bla*_IMP_ nor *bla*_VIM_ was detected in all isolates. Comparative analysis revealed distinct patterns of carbapenemase gene distribution between CR-HvKP and CR-non-HvKP isolates (Table S4). The prevalence of *bla*_KPC−2_ was significantly higher in CR-HvKP isolates compared to CR-non-HvKP isolates (91.7% vs. 28.1%, *p* < 0.001). Strikingly, both *bla*_NDM−5_ and *bla*_OXA−48_ were exclusively detected in CR-non-HvKP isolates (0% vs. 56.3%, *p* < 0.001; 0% vs. 25.0%, *p* = 0.001, respectively).

As shown in Table 3, most carbapenemase-producing isolates exhibited MIC ≥ 16 µg/mL for imipenem and meropenem, confirming high-level resistance regardless of genotype. No significant differences were observed between *bla*_KPC−2_, *bla*_NDM−5_, and *bla*_OXA−48_ groups (*p* > 0.05). The VITEK^®^ 2 system’s upper MIC limit (16 µg/mL) prevented further stratification of high-level resistance. All MIC data are provided in Table S5.

**Table 3:** Relationship between MIC values ​​and specific carbapenemases

Antibiotic	MIC Range (µg/mL)	MIC ≥ 16 µg/mL (%)	*bla*_KPC−2_ (*n* = 42)	*bla*_NDM−5_ (*n* = 18)	*bla*_OXA−48_ (*n* = 8)	*p*-value
Imipenem^a^	8-≥ 16	97.1%	97.6% ≥ 16	94.4% ≥ 16	87.5% ≥ 16	0.21
Meropenem^a^	8-≥ 16	97.1%	100% ≥ 16	100% ≥ 16	100% ≥ 16	/

^a^Actual MIC of most strains exceeds detection limit (16 μg/mL)

### MLST and correlation between STs and KLs

**Fig. 3:**
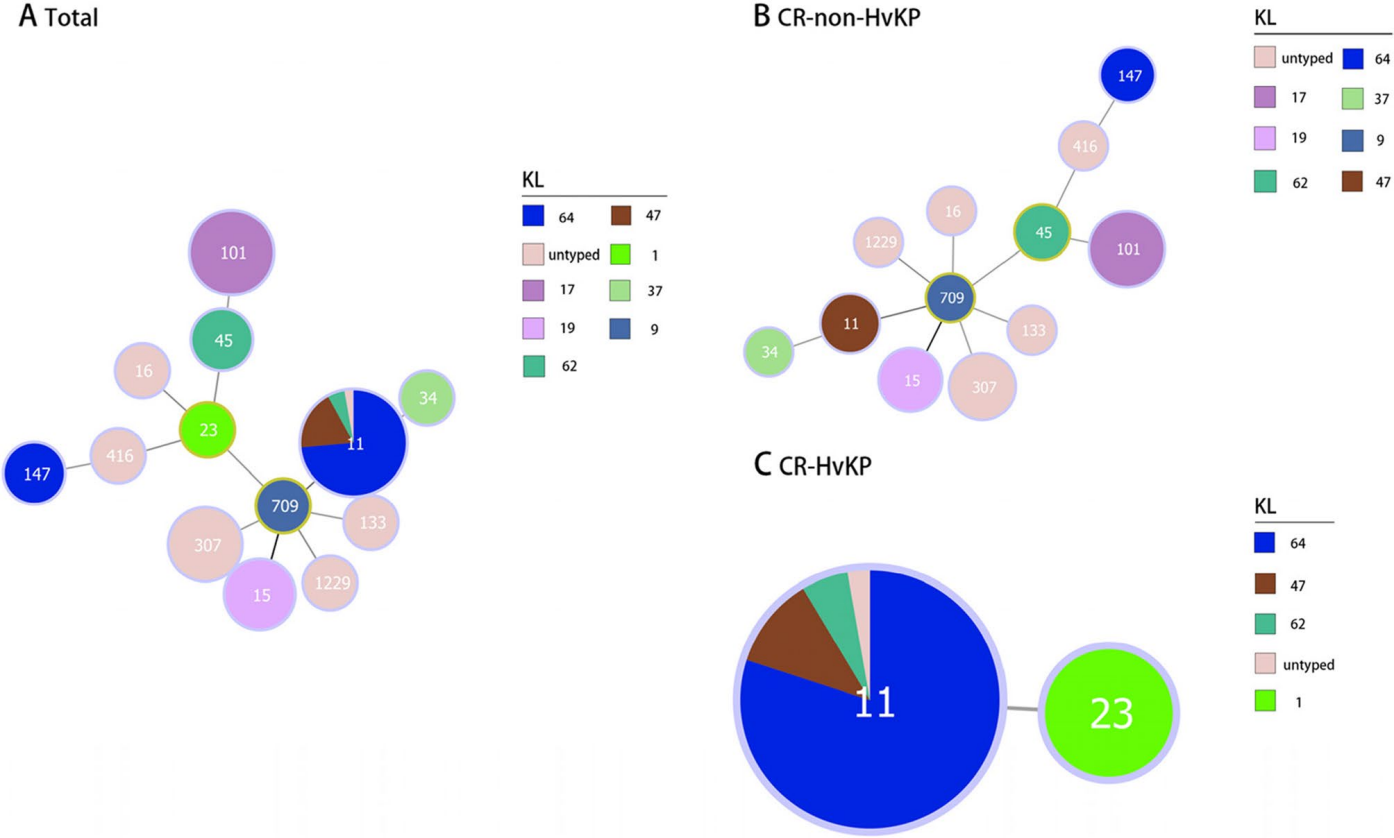
Minimum spanning tree based on the alleles of seven housekeeping genes illustrating the relationship between STs and KLs in CR-HvKP strains and CR-non-HvKP strains. Numbers indicate the different STs. ST, Sequence type; KL, K locus

Molecular typing revealed 13 distinct STs among the 68 CRKP isolates (Fig. 2). ST11 emerged as the predominant lineage (38/68, 55.9%), forming a well-defined cluster, while other STs showed a more dispersed distribution, such as ST101 (10/68, 14.7%), ST307 (5/68, 7.4%), and ST15 (4/68, 5.9%). This distribution pattern shows the prevalence of ST11 among CR-HvKP isolates and ST101 among CR-non-HvKP isolates in our cohort. Phylogenetic analysis identified a ST709 CR-non-HvKP isolate as a single-locus variant (SLV) of the four ST15 CR-non-HvKP isolates, while limited phylogenetic relationships were observed among the remaining STs. Minimum spanning tree analysis demonstrated a strong association between specific STs and KLs, particularly ST11-KL64 and ST101-KL17 (Fig. 3). Of particular interest was the identification of both the prevalent ST11-KL64 CR-HvKP isolate and a single ST23-KL1 CR-HvKP isolate, suggesting the potential existence of distinct evolutionary trajectories in CR-HvKP development.

### Risk factors of patients with CR-HvKP infection

Univariate analysis revealed three variables with significant associations: ICU admission (*p* = 0.018), respiratory department admission (*p* = 0.029), and prior carbapenem exposure (*p* = 0.023). Based on clinical relevance to CR-HvKP infection, ICU admission and carbapenem exposure were selected for subsequent multivariate logistic regression analysis. Although neither variable emerged as an independent risk factor for CR-HvKP infection in the multivariate model, ICU admission approached statistical significance (OR = 2.939, *p* = 0.056) (Table 4), suggesting its potential role as a clinically relevant risk factor that warrants further investigation.

**Table 4:** Univariate and multivariate logistic regression analysis of patients with CR-HvKP infections

Variables	Univariate analysis		Multivariable analysis	
	OR (95% CI)	*p*-value	OR (95% CI)	*p*-value
ICU	3.536 (1.214–10.297)	**0.021**^a^	2.939 (0.972–8.888)	0.056
Carbapenems	3.300 (1.160–9.384)	**0.025**^a^	2.718 (0.917–8.058)	0.071

^a^Bold indicates *p*-value < 0.05

*ICU* intensive care unit, *OR* Odds ratio

## Discussion

In recent years, there has been a steady increase in global reports of CR-HvKP [25]. This pathogen poses significant clinical challenges due to its potent colonization capacity, multidrug resistance profile, and heightened pathogenicity, characteristics that facilitate its dissemination across both clinical and environmental settings [15]. In this study, we systematically screened clinical CRKP isolates to compare the clinical and microbiological characteristics between CR-HvKP and CR-non-HvKP strains. Our findings demonstrate the dominance of ST11-KL64 CR-HvKP clones carrying *bla*_KPC−2_ and *iucA* in our hospital, highlighting an emerging local threat.

It is well-established that HvKP constitutes the primary etiological agent of liver abscesses [26]. However, our analysis revealed that among 32 CR-HvKP-infected patients, the predominant clinical manifestation was pneumonia (65.6%, 21/32), with only 6.3% (2/32) presenting with liver abscesses, which is similar to previous study [27]. This observation suggests that CR-HvKP may be associated with extrahepatic infectious manifestations, including pneumonia, urinary tract infections, and bacteremia [28]. Notably, CR-HvKP-infected patients demonstrated significantly higher ICU admission rates (*p* = 0.018) and prior carbapenem exposure (*p* = 0.023) compared to CR-non-HvKP cases, potentially implicating ICU environments as reservoirs for multidrug-resistant pathogen transmission [4]. Although clinicians may opt to administer carbapenems in combination with other antimicrobial agents for CRKP infections, such as tigecycline, this therapeutic approach may inadvertently promote the dissemination of CR-HvKP strains [29]. Additionally, our multivariate analysis did not identify ICU exposure or carbapenem use as independent risk factors for CR-HvKP acquisition, however the borderline significance observed for ICU residence rate (OR = 2.939, *p* = 0.056) warrants attention. We strongly advocate for enhanced surveillance of ICU environments and rigorous antimicrobial stewardship programs, particularly regarding judicious carbapenem utilization.

Comparative analysis revealed a notable discrepancy between phenotypic and genotypic virulence assessments. While prior study has established the string test as a conventional method for identifying HvKP [8], our findings demonstrated only 22.2% concordance (8/36) between string test results and virulence gene profiling, which resembled other study [20]. This suggests that morphological traits such as colony appearance may not consistently correlate with genotypic virulence in CR-HvKP, emphasizing the necessity of molecular diagnostics for accurate identification. This phenotypic-genotypic discordance was particularly evident in our CR-HvKP, where one isolate exhibited hypermucoviscosity phenotype despite testing negative for both *rmpA* and *rmpA2*, with an additional four strains showing complete absence of both hypermucoviscosity characteristics and these regulatory genes. Interestingly, the universal presence of *iucA* across all CR-HvKP isolates (36/36) suggests its potential as a defining virulence determinant in CR-HvKP. This observation aligns with established mechanisms where aerobactin biosynthesis mediated by *iucA* significantly enhances bacterial iron acquisition and pathogenicity [30]. Our data support the hypothesis that *iucA* may compensate for the absence of other virulence regulators in CR-HvKP strains, potentially explaining their maintained pathogenicity despite *rmpA*/*rmpA2* deficiencies [31]. Moreover, our results showed that *iroB* was absent in most CR-HvKP, suggesting that *iroB* deletion promoted cross-regional transmission of CR-HvKP [32]. These findings collectively suggest three critical implications: First, current phenotypic screening methods require supplementation with molecular methods to identify for CR-HvKP. Second, the therapeutic targeting of aerobactin synthesis through novel inhibitors emerges as a promising strategy against CR-HvKP infections. Third, the acquisition or loss of virulence-associated genes can drive the clonal expansion and dissemination of CR-HvKP strains.

Our findings demonstrate a concerning multidrug-resistant phenotype among all analyzed strains, exhibiting resistance to major antibiotic classes including cephalosporins, β-lactam/β-lactamase inhibitor, carbapenems, fluoroquinolones, and aminoglycosides. Carbapenemase production (approximately 90%) was identified as the principal mechanism underlying the extensive multidrug resistance in CRKP strains, with KPC enzymes being predominant (60.3%), followed by NDM (26.5%) [33]. Notably, *bla*_KPC−2_ exhibited remarkably high prevalence (91.7%) in CR-HvKP isolates, while *bla*_NDM−5_ and *bla*_OXA−48_ were exclusively detected in CR-non-HvKP strains. No isolates carried any of *bla*_IMP_ or *bla*_VIM_. This distinct distribution pattern appears attributable to genetic context: *bla*_KPC−2_ was predominantly located on *IncF*-type plasmids, which frequently co-harbor virulence determinants such as *iucA* [34]. This co-localization facilitates concurrent evolution of multidrug resistance and hypervirulence through horizontal gene transfer. Conversely, *bla*_NDM−5_ was primarily associated with *IncX3* plasmids demonstrating enhanced stability in CR-non-HvKP genetic backgrounds [35]. While tigecycline (91.2% susceptibility) and polymyxin B (85.3% susceptibility) retained significant in vitro activity, their clinical utility requires judicious evaluation. The nephrotoxicity associated with polymyxins and emerging tigecycline resistance mechanisms necessitate strict adherence to antimicrobial stewardship principles. Empirical therapy for CR-HvKP infections should be guided by comprehensive antimicrobial susceptibility testing.

As for high-risk epidemic clones, our study primarily focused on the ST11-KL64 CR-HvKP clone, which has been epidemiologically linked to nosocomial infection outbreaks in East Asia, particularly in China [36, 37]. Compared to CR-non-HvKP strains dominated by ST101-KL17, the ST11-KL64 CR-HvKP represents a convergent evolutionary trajectory conferring selective advantages through integration of virulence plasmids and resistance plasmids, forming dual-threat clones with enhanced pathogenic potential [38]. While ST23-KL1 clones have historically demonstrated carbapenem susceptibility, recent literature has documented the emergence of ST23-KL1 CR-HvKP [39]. In line with these findings, our study similarly identified a ST23-KL1 CR-HvKP strain, notably lacking detectable carbapenemase-encoding genes. This observation suggests potential acquisition of alternative resistance mechanisms under antibiotic selection pressure, possibly involving efflux pump activation, targets modification, or other adaptive membrane alterations [40]. Collectively, ST11-KL64 and ST23-KL1 represent divergent evolutionary pathways in CR-HvKP development. Additionally, our analysis revealed seven ST11-KL47 strains distributed across both CR-HvKP and CR-non-HvKP. The ST11-KL47 CR-HvKP isolates demonstrated significant ICU clustering, suggesting ICU admission may constitute an independent risk factor for CR-HvKP acquisition.

To date, this study represents the first comprehensive clinical and molecular epidemiological investigation of CR-HvKP in Western China. Nevertheless, several limitations warrant consideration. Firstly, although *wzi* gene sequencing was employed for KL capsule serotyping, accurate typing could not be achieved for a small subset of strains. Future investigations may necessitate the implementation of whole genome sequencing approaches. Secondly, while we utilized an integrated approach to characterize virulence factors in CR-HvKP, our findings lack validation through experimental models, such as Galleria mellonella or murine infection model. Recently, the results of study by Kochan et al. suggested that CR-HvKP strains may have unexpectedly low virulence [41]. Thirdly, this study did not investigate molecular adaptations of CR-HvKP under antibiotic pressure. Future work using transcriptomic approaches could elucidate mechanisms of resistance evolution in response to antimicrobial therapies. Finally, the present study is constrained by its single-center design, limited sample size, and cross-sectional nature within a one-year timeframe. More extensive, longitudinal surveillance studies are essential to generate comprehensive data regarding the clinical and molecular characteristics of CR-HvKP strains.

In conclusion, CR-HvKP has emerged as an increasingly prevalent pathogen in healthcare settings, with particular concern centered on the high-risk clone ST11-KL64 CR-HvKP harboring *bla*_KPC−2_ and *iucA*. Given the significant challenges posed by the concurrent evolution of virulence and antimicrobial resistance in these high-risk CR-HvKP clones, enhanced molecular surveillance programs and refined infection prevention and control strategies are imperative for effective management of this emerging threat.

## Supplementary Information

Supplementary Material 1.

Supplementary Material 2.

## Data Availability

Data is provided within the manuscript or supplementary information files.

## Abbreviations

CRKP: Carbapenem-resistant *K. pneumoniae*
HvKP: Hypervirulent *K. pneumoniae*
WHO: World Health Organization
CR-HvKP: Carbapenem-resistant hypervirulent *K. pneumoniae*
MALDI-TOF MS: Matrix-Assisted Laser Desorption/Ionization-Time of Flight Mass Spectrometry
FDA: Food and Drug Administration
MLST: Multilocus sequence typing
STs: Sequence types
iTOL: Interactive Tree of Life
ICU: Intensive care unit
SLV: Single-locus variant
