# Supplementary material for: Emergence of ST11-KL64 carbapenem-resistant hypervirulent Klebsiella Pneumoniae isolates harboring blaKPC−2 and iucA from a tertiary teaching hospital in Western China
Source: BMC Infect Dis. 2025 Jul 1;25:880. doi: 10.1186/s12879-025-11241-6 (PMC12220110; doi:10.1186/s12879-025-11241-6)
Supplement: Supplementary file 2 — Supplementary Material 2. [file 12879_2025_11241_MOESM2_ESM.docx]

**Table S1** Amplification primers used in this study.

| **Genes** | **Primer sequence (5′-3′)** | **Annealing temperature(◦C)** | **Size (bp)** |
| --- | --- | --- | --- |
| **Virulence genes** |  |  |  |
| *rmpA* | F: ATGTGGCTTGACGTTTCGGGGG | 55 | 160 |
|  | R: GCCGTGGATAATGGTTTACAATTCGGC |  |  |
| *rmpA2* | F: GGATGTGGCTTGACATTTCGGGGG | 55 | 227 |
|  | R: TTCATGGATGCCCTCCCTCCTG |  |  |
| *iroB* | F: ATCTCATCATCTACCCTCCGCTC | 58 | 235 |
|  | R: GGTTCGCCGTCGTTTTCAA |  |  |
| *iucA* | F: AATCAATGGCTATTCCCGCTG | 56 | 239 |
|  | R: CGCTTCACTTCTTTCACTGACAGG |  |  |
| *peg-344* | F: AAAGGACAGAAAGCCAGTG | 53 | 411 |
|  | R: CAATGACGAGGGGGATAATC |  |  |
| **Carbapenemase genes** |  |  |  |
| *bla*_KPC_ | F: ATGTCACTGTATCGCCGTCT | 55 | 893 |
|  | R: TTTTCAGAGCCTTACTGCCC |  |  |
| *bla*_NDM_ | F: ATGGAATTGCCCAATATTATGC | 55 | 813 |
|  | R: TCAGCGCAGCTTGTCGG |  |  |
| *bla*_OXA-48_ | F: GCGTGGTTAAGGATGAACAC | 56 | 438 |
|  | R: CATCAAGTTCAACCCAACCG |  |  |
| *bla*_VIM_ | F: GGTCGCATATCGCAACGCAGT | 55 | 636 |
|  | R: CGGCGACTGAGCGATTTTTG |  |  |
| *bla*_IMP_ | F: ATGAGCAAGTTATCTGTATTCTTTAT | 55 | 741 |
|  | R: TTAGTTGCTTAGTTTTGATGGTTT |  |  |
| **Housekeeping genes** |  |  |  |
| *rpoB* | F: GGCGAAATGGCWGAGAACCA | 50 | 1075 |
|  | R: GAGTCTTCGAAGTTGTAACC |  |  |
| *gapA* | F: TGAAATATGACTCCACTCACGG | 60 | 662 |
|  | R: CTTCAGAAGCGGCTTTGATGGCTT |  |  |
| *mdh* | F: CCCAACTCGCTTCAGGTTCAG | 50 | 756 |
|  | R: CCGTTTTTCCCCAGCAGCAG |  |  |
| *pgi* | F: GAGAAAAACCTGCCTGTACTGCTGGC | 50 | 718 |
|  | R: CGCGCCACGCTTTATAGCGGTTAAT |  |  |
| *phoE* | F: ACCTACCGCAACACCGACTTCTTCGG | 50 | 602 |
|  | R: TGATCAGAACTGGTAGGTGAT |  |  |
| *infB* | F: CTCGCTGCTGGACTATATTCG | 50 | 462 |
|  | R: CGCTTTCAGCTCAAGAACTTC |  |  |
| *tonB* | F: CTTTATACCTCGGTACATCAGGTT | 45 | 539 |
|  | R: ATTCGCCGGCTGRGCRGAGAG |  |  |
| **Capsular serotype** |  |  |  |
| *wzi* | F: GTGCCGCGAGCGCTTTCTATCTTGGTATTCC | 55 | 581 |
|  | R: GAGAGCCACTGGTTCCAGAACTTCACCGC |  |  |

**Table S2** Antimicrobial resistance rate of 68 CRKP strains in this study.

| **Antimicrobial agents** | **CR-HvKP (*n*, %)**  **(*n* = 36)** | **CR-non-HvKP (*n*, %)**  **(*n* = 32)** | ***p*-value** |
| --- | --- | --- | --- |
| AMP | 36 (100) | 32 (100) | / |
| SAM | 36 (100) | 32 (100) | / |
| TZP | 36 (100) | 32 (100) | / |
| CEF | 36 (100) | 32 (100) | / |
| CAZ | 36 (100) | 32 (100) | / |
| FEP | 36 (100) | 31 (96.9) | 0.285 |
| SCF | 36 (100) | 29 (90.6) | 0.171 |
| IPM | 36 (100) | 32 (100) | / |
| MEM | 36 (100) | 32 (100) | / |
| ATM | 36 (100) | 31 (96.9) | 0.285 |
| LVX | 36 (100) | 31 (96.9) | 0.285 |
| CIP | 36 (100) | 31 (96.9) | 0.285 |
| SXT | 34 (94.4) | 25 (78.1) | **0.047^a^** |
| TET | 36 (100) | 29 (90.6) | 0.06 |
| TGC | 0 (0) | 0 (0) | / |
| AK | 34 (94.4) | 17 (53.1) | **< 0.001^a^** |
| TOB | 34 (94.4) | 24 (75.0) | **0.046^a^** |
| POL | 8 (22.2) | 2 (6.3) | 0.089 |

Data are presented as frequencies and percentages, unless otherwise stated.

^a^ Bold indicates *p*-value < 0.05.

AMP, ampicillin; ASM, ampicillin/sulbactam; TZP, piperacillin/tazobactam; CEF, cefalotin; CAZ, ceftazidime; FEP, cefepime; SCF, cefoperazone/sulbactam; IPM, imipenem; MEM, meropenem; ATM, aztreonam; CIP, ciprofloxacin; LVX, levofloxacin; AK, amikacin; TOB, tobramycin; SXT, trimethoprim/sulfamethoxazole; TET, tetracycline; TGC, tigecycline; POL, polymyxin B.

**Table S3** Carbapenemase inhibitor enhancement test on 68 CRKP strains for carbapenemase production.

| **strains** | **Control (mm)** | **EDTA (mm)** | **APB (mm)** | **EDTA+APB (mm)** | **Phenotypic results** | **Carbapenemase** |
| --- | --- | --- | --- | --- | --- | --- |
| KP-1 | 11 | 16 | 12 | 16 | 0 | NDM-5 |
| KP-3 | 6 | 9 | 7 | 15 | 3 | KPC-2+NDM-13 |
| KP-4 | 6 | 6 | 13 | 15 | 1 | KPC-2 |
| KP-7 | 6 | 19 | 8 | 19 | 0 | NDM-5 |
| KP-9 | 12 | 21 | 13 | 21 | 0 | NDM-5 |
| KP-10 | 6 | 6 | 14 | 15 | 1 | KPC-2 |
| KP-11 | 10 | 18 | 10 | 18 | 0 | NDM-5 |
| KP-12 | 6 | 8 | 17 | 17 | 1 | KPC-2 |
| KP-15 | 10 | 11 | 10 | 11 | 2 | KPC-14 |
| KP-16 | 6 | 8 | 17 | 14 | 1 | KPC-2 |
| KP-17 | 8 | 17 | 10 | 17 | 0 | NDM-5 |
| KP-20 | 9 | 10 | 17 | 18 | 1 | KPC-2 |
| KP-21 | 7 | 15 | 10 | 14 | 0 | NDM-5 |
| KP-23 | 6 | 21 | 6 | 23 | 0 | NDM-5 |
| KP-24 | 13 | 13 | 22 | 23 | 1 | KPC-2 |
| KP-25 | 10 | 18 | 10 | 19 | 0 | NDM-5 |
| KP-26 | 9 | 10 | 20 | 21 | 1 | KPC-2 |
| KP-27 | 6 | 7 | 15 | 17 | 1 | KPC-2 |
| KP-28 | 6 | 6 | 17 | 18 | 1 | KPC-2 |
| KP-29 | 6 | 6 | 16 | 17 | 1 | KPC-2 |
| KP-31 | 6 | 8 | 17 | 28 | 1 | KPC-2 |
| KP-32 | 14 | 15 | 20 | 21 | 1 | KPC-2 |
| KP-36 | 10 | 12 | 18 | 20 | 1 | KPC-2 |
| KP-39 | 9 | 10 | 11 | 12 | 2 | KPC-14 |
| KP-40 | 6 | 15 | 6 | 15 | 0 | NDM-5 |
| KP-44 | 9 | 16 | 9 | 17 | 0 | NDM-5 |
| KP-45 | 6 | 6 | 17 | 17 | 1 | KPC-2 |
| KP-53 | 15 | 21 | 19 | 24 | 0 | NDM-5 |
| KP-54 | 11 | 19 | 9 | 20 | 0 | NDM-5 |
| KP-55 | 7 | 11 | 20 | 20 | 1 | \ |
| KP-57 | 15 | 16 | 22 | 20 | 1 | KPC-2 |
| KP-60 | 6 | 8 | 7 | 14 | 3 | KPC-2+NDM-13 |
| KP-61 | 15 | 20 | 15 | 20 | 0 | NDM-5 |
| KP-62 | 7 | 8 | 18 | 18 | 1 | KPC-2 |
| KP-63 | 7 | 19 | 7 | 20 | 0 | NDM-5 |
| KP-64 | 11 | 20 | 11 | 21 | 0 | NDM-5 |
| KP-65 | 6 | 8 | 18 | 19 | 1 | KPC-2 |
| KP-66 | 10 | 9 | 17 | 17 | 1 | KPC-2 |
| KP-69 | 7 | 8 | 17 | 17 | 1 | KPC-2 |
| KP-5 | 6 | 7 | 6 | 17 | 3 | KPC-2+NDM-13 |
| KP-6 | 10 | 10 | 19 | 19 | 1 | KPC-2 |
| KP-70 | 13 | 15 | 15 | 16 | 2 | \ |
| KP-71 | 6 | 6 | 15 | 15 | 1 | KPC-2 |
| KP-72 | 6 | 6 | 14 | 14 | 1 | KPC-2 |
| KP-73 | 6 | 6 | 14 | 14 | 1 | KPC-2 |
| KP-74 | 6 | 6 | 13 | 13 | 1 | KPC-2 |
| KP-75 | 6 | 7 | 13 | 13 | 1 | KPC-2 |
| KP-76 | 6 | 6 | 11 | 12 | 1 | KPC-2 |
| KP-77 | 6 | 6 | 10 | 10 | 2 | \ |
| KP-78 | 6 | 6 | 13 | 13 | 1 | KPC-2 |
| KP-80 | 6 | 6 | 13 | 13 | 1 | KPC-2 |
| KP-81 | 6 | 6 | 13 | 15 | 1 | KPC-2 |
| KP-82 | 6 | 6 | 15 | 16 | 1 | KPC-2 |
| KP-84 | 11 | 12 | 13 | 14 | 2 | \ |
| KP-87 | 6 | 6 | 13 | 14 | 1 | KPC-2 |
| KP-88 | 12 | 13 | 19 | 19 | 1 | KPC-2 |
| KP-90 | 7 | 9 | 15 | 16 | 1 | KPC-2 |
| KP-91 | 13 | 15 | 13 | 16 | 2 | \ |
| KP-92 | 6 | 11 | 6 | 12 | 0 | NDM-5 |
| KP-94 | 6 | 6 | 14 | 14 | 1 | KPC-2 |
| KP-96 | 6 | 6 | 13 | 13 | 1 | KPC-2 |
| KP-97 | 6 | 6 | 13 | 14 | 1 | KPC-2 |
| KP-98 | 6 | 6 | 12 | 12 | 1 | KPC-2 |
| KP-99 | 6 | 11 | 7 | 12 | 0 | NDM-5 |
| KP-100 | 6 | 6 | 14 | 15 | 1 | KPC-2 |
| KP-101 | 6 | 11 | 7 | 12 | 0 | NDM-5 |
| KP-102 | 12 | 11 | 13 | 13 | 2 | \ |
| KP-103 | 6 | 6 | 13 | 13 | 1 | KPC-2 |

0 means metallo-β-lactamase, 1 means class A β-lactamase, 2 means neither 0 nor 1, 3 means both 0 and 1.

APB, 3-aminophenylboronic acid; EDTA, Ethylene Diamine Tetraacetic Acid.

**Table S4** Distribution of K types and carbapenemase-associated genes among 68 CRKP isolates.

| **Variables** | **CR-HvKP (*n*, %)**  **(*n* = 36)** | **CR-non-HvKP (*n*, %)**  **(*n* = 32)** | ***p*-value** |
| --- | --- | --- | --- |
| KL64 | 28 (77.8) | 2 (6.3) | **< 0.001^a^** |
| KL17 | 0 (0) | 10 (31.3) | **< 0.001^a^** |
| KL19 | 0 (0) | 4 (12.5) | **0.044^a^** |
| Untyped | 1 (2.8) | 9 (28.1) | **0.005^a^** |
| KL47 | 4 (11.1) | 3 (9.4) | 1.000 |
| KL1 | 1 (2.8) | 0 (0) | 1.000 |
| KL9 | 0 (0) | 1 (3.1) | 0.471 |
| KL37 | 0 (0) | 1 (3.1) | 0.471 |
| KL62 | 2 (5.6) | 2 (6.3) | 1.000 |
| *bla*_KPC-2_ | 33 (91.7) | 9 (28.1) | **< 0.001^a^** |
| *bla*_NDM-5_ | 0 (0) | 18 (56.3) | **< 0.001^a^** |
| *bla*_OXA-48_ | 0 (0) | 8 (25.0) | **0.001^a^** |
| *bla*_KPC-14_ | 2 (5.6) | 0 (0) | 0.494 |
| *bla*_NDM-13_ | 3 (8.3) | 0 (0) | 0.241 |
| *bla*_IMP_ | 0 (0) | 0 (0) | / |
| *bla*_VIM_ | 0 (0) | 0 (0) | / |

Data are presented as frequencies and percentages, unless otherwise stated.

^a^ Bold indicates *p*-value < 0.05.

KL, K locus.

**Table S5** MIC values ​​and MIC range of 68 CRKP strains to different antibiotics.

| **strains** | **AMP** | **SAM** | **TZP** | **CEF** | **CAZ** | **FEP** | **SCF** | **IPM** | **MEM** | **ATM** | **LVX** | **CIP** | **SXT** | **TET** | **TGC** | **AK** | **TOB** | **POL** |
| --- | --- | --- | --- | --- | --- | --- | --- | --- | --- | --- | --- | --- | --- | --- | --- | --- | --- | --- |
| KP-1 | >=32 | >=32 | >=128 | >=64 | >=64 | 32 | >=64 | 8 | >=16 | >=64 | >=8 | >=4 | >=320 | >=16 | 1 | >=64 | >=16 | 0.5 |
| KP-3 | >=32 | >=32 | >=128 | >=64 | >=64 | >=64 | >=64 | >=16 | >=16 | >=64 | >=8 | >=4 | >=320 | >=16 | 1 | >=64 | >=16 | 0.5 |
| KP-4 | >=32 | >=32 | >=128 | >=64 | >=64 | >=64 | >=64 | >=16 | >=16 | >=64 | >=8 | >=4 | >=320 | >=16 | 4 | >=64 | >=16 | 32 |
| KP-7 | >=32 | >=32 | >=128 | >=64 | >=64 | 32 | >=64 | >=16 | >=16 | >=64 | >=8 | >=4 | <=20 | >=16 | 2 | <=2 | <=1 | 0.5 |
| KP-9 | >=32 | >=32 | >=128 | >=64 | >=64 | 32 | >=64 | >=16 | >=16 | >=64 | >=8 | >=4 | >=320 | >=16 | 1 | >=64 | >=16 | 0.5 |
| KP-10 | >=32 | >=32 | >=128 | >=64 | >=64 | 32 | >=64 | >=16 | >=16 | >=64 | >=8 | >=4 | >=320 | >=16 | 2 | >=64 | >=16 | 0.5 |
| KP-11 | >=32 | >=32 | >=128 | >=64 | >=64 | 16 | >=64 | >=16 | >=16 | 4 | 4 | 2 | <=20 | 4 | 0.25 | <=2 | <=1 | 1 |
| KP-12 | >=32 | >=32 | >=128 | >=64 | >=64 | 32 | >=64 | >=16 | >=16 | >=64 | >=8 | >=4 | >=320 | >=16 | 2 | >=64 | >=16 | 0.5 |
| KP-15 | >=32 | >=32 | >=128 | >=64 | >=64 | >=64 | >=64 | >=16 | >=16 | >=64 | >=8 | >=4 | >=320 | >=16 | 2 | >=64 | >=16 | 0.5 |
| KP-16 | >=32 | >=32 | >=128 | >=64 | >=64 | >=64 | >=64 | >=16 | >=16 | >=64 | >=8 | >=4 | >=320 | >=16 | 4 | >=64 | >=16 | 16 |
| KP-17 | >=32 | >=32 | >=128 | >=64 | >=64 | 32 | >=64 | >=16 | >=16 | >=64 | >=8 | >=4 | >=320 | >=16 | 2 | >=64 | >=16 | 0.5 |
| KP-20 | >=32 | >=32 | >=128 | >=64 | >=64 | >=64 | >=64 | >=16 | >=16 | >=64 | >=8 | >=4 | >=320 | >=16 | 2 | >=64 | >=16 | 32 |
| KP-21 | >=32 | >=32 | >=128 | >=64 | >=64 | 32 | >=64 | >=16 | >=16 | >=64 | >=8 | >=4 | >=320 | >=16 | 2 | >=64 | >=16 | 0.5 |
| KP-23 | >=32 | >=32 | >=128 | >=64 | >=64 | 32 | >=64 | >=16 | >=16 | >=64 | >=8 | >=4 | 160 | >=16 | 2 | 8 | >=16 | 0.5 |
| KP-24 | >=32 | >=32 | >=128 | >=64 | >=64 | 16 | >=64 | >=16 | >=16 | >=64 | >=8 | >=4 | >=320 | >=16 | 4 | >=64 | >=16 | 0.5 |
| KP-25 | >=32 | >=32 | >=128 | >=64 | >=64 | 32 | >=64 | >=16 | >=16 | >=64 | >=8 | >=4 | >=320 | >=16 | 0.5 | <=2 | 8 | 32 |
| KP-26 | >=32 | >=32 | >=128 | >=64 | >=64 | 32 | >=64 | >=16 | >=16 | >=64 | >=8 | >=4 | >=320 | >=16 | 2 | >=64 | >=16 | 16 |
| KP-27 | >=32 | >=32 | >=128 | >=64 | >=64 | 32 | >=64 | >=16 | >=16 | >=64 | >=8 | >=4 | >=320 | >=16 | 1 | >=64 | >=16 | 0.5 |
| KP-28 | >=32 | >=32 | >=128 | >=64 | >=64 | 32 | >=64 | >=16 | >=16 | >=64 | >=8 | >=4 | >=320 | >=16 | 1 | >=64 | >=16 | 0.5 |
| KP-29 | >=32 | >=32 | >=128 | >=64 | >=64 | 32 | >=64 | >=16 | >=16 | >=64 | >=8 | >=4 | >=320 | >=16 | 0.25 | >=64 | >=16 | 0.5 |
| KP-31 | >=32 | >=32 | >=128 | >=64 | >=64 | 32 | >=64 | >=16 | >=16 | >=64 | >=8 | >=4 | <=20 | >=16 | 2 | >=64 | >=16 | 4 |
| KP-32 | >=32 | >=32 | >=128 | >=64 | >=64 | 32 | >=64 | >=16 | >=16 | >=64 | >=8 | >=4 | >=320 | >=16 | 2 | 8 | >=16 | 0.5 |
| KP-36 | >=32 | >=32 | >=128 | >=64 | 32 | 16 | >=64 | >=16 | >=16 | >=64 | >=8 | >=4 | >=320 | >=16 | 2 | >=64 | >=16 | 0.5 |
| KP-39 | >=32 | >=32 | >=128 | >=64 | >=64 | 32 | >=64 | >=16 | 8 | >=64 | >=8 | >=4 | >=320 | >=16 | 2 | >=64 | >=16 | 0.5 |
| KP-40 | >=32 | >=32 | >=128 | >=64 | >=64 | 32 | >=64 | >=16 | >=16 | >=64 | >=8 | >=4 | >=320 | >=16 | 2 | >=64 | >=16 | 0.5 |
| KP-44 | >=32 | >=32 | >=128 | >=64 | >=64 | 32 | >=64 | >=16 | >=16 | >=64 | >=8 | >=4 | >=320 | >=16 | 2 | >=64 | >=16 | 0.5 |
| KP-45 | >=32 | >=32 | >=128 | >=64 | >=64 | >=64 | >=64 | >=16 | >=16 | >=64 | >=8 | >=4 | 80 | >=16 | 1 | <=2 | <=1 | 0.5 |
| KP-53 | >=32 | >=32 | >=128 | >=64 | >=64 | >=64 | >=64 | >=16 | >=16 | >=64 | >=8 | >=4 | >=320 | >=16 | 4 | <=2 | >=16 | 0.5 |
| KP-54 | >=32 | >=32 | >=128 | >=64 | >=64 | >=64 | >=64 | >=16 | >=16 | >=64 | >=8 | >=4 | >=320 | >=16 | 4 | <=2 | 8 | 2 |
| KP-55 | >=32 | >=32 | >=128 | >=64 | >=64 | >=64 | >=64 | >=16 | >=16 | >=64 | >=8 | >=4 | <=20 | >=16 | 2 | <=2 | <=1 | 0.5 |
| KP-57 | >=32 | >=32 | >=128 | >=64 | 32 | 32 | >=64 | 8 | >=16 | >=64 | >=8 | >=4 | >=320 | >=16 | 1 | <=2 | >=16 | 0.5 |
| KP-60 | >=32 | >=32 | >=128 | >=64 | >=64 | >=64 | >=64 | >=16 | >=16 | >=64 | >=8 | >=4 | >=320 | >=16 | 2 | >=64 | >=16 | 0.5 |
| KP-61 | >=32 | >=32 | >=128 | >=64 | >=64 | >=64 | 32 | >=16 | >=16 | >=64 | >=8 | >=4 | >=320 | >=16 | 1 | 4 | >=16 | 1 |
| KP-62 | >=32 | >=32 | >=128 | >=64 | >=64 | 32 | >=64 | >=16 | >=16 | >=64 | >=8 | >=4 | >=320 | >=16 | 1 | >=64 | >=16 | 32 |
| KP-63 | >=32 | >=32 | >=128 | >=64 | >=64 | 32 | >=64 | >=16 | >=16 | >=64 | >=8 | >=4 | 80 | >=16 | 2 | <=2 | <=1 | 0.5 |
| KP-64 | >=32 | >=32 | >=128 | >=64 | >=64 | >=64 | >=64 | >=16 | >=16 | >=64 | >=8 | >=4 | >=320 | >=16 | 2 | >=64 | >=16 | 0.5 |
| KP-65 | >=32 | >=32 | >=128 | >=64 | >=64 | 32 | >=64 | >=16 | >=16 | >=64 | >=8 | >=4 | <=20 | >=16 | 1 | >=64 | >=16 | 0.5 |
| KP-66 | >=32 | >=32 | >=128 | >=64 | >=64 | >=64 | >=64 | >=16 | >=16 | >=64 | >=8 | >=4 | >=320 | >=16 | 2 | >=64 | >=16 | 0.5 |
| KP-69 | >=32 | >=32 | >=128 | >=64 | >=64 | >=64 | >=64 | >=16 | >=16 | >=64 | >=8 | >=4 | >=320 | >=16 | 0.5 | >=64 | >=16 | 0.5 |
| KP-5 | >=32 | >=32 | >=128 | >=64 | >=64 | >=64 | >=64 | >=16 | >=16 | >=64 | >=8 | >=4 | >=320 | >=16 | 2 | >=64 | >=16 | 1 |
| KP-6 | >=32 | >=32 | >=128 | >=64 | >=64 | >=64 | >=64 | >=16 | >=16 | >=64 | >=8 | >=4 | 40 | >=16 | 1 | 4 | >=16 | 1 |
| KP-70 | >=32 | >=32 | >=128 | >=64 | 16 | >=64 | 16 | >=16 | >=16 | >=64 | >=8 | >=4 | <=20 | 4 | 2 | >=64 | >=16 | 1 |
| KP-71 | >=32 | >=32 | >=128 | >=64 | >=64 | >=64 | >=64 | >=16 | >=16 | >=64 | >=8 | >=4 | >=320 | >=16 | 1 | >=64 | >=16 | 1 |
| KP-72 | >=32 | >=32 | >=128 | >=64 | >=64 | >=64 | >=64 | >=16 | >=16 | >=64 | >=8 | >=4 | >=320 | >=16 | 4 | >=64 | >=16 | 1 |
| KP-73 | >=32 | >=32 | >=128 | >=64 | >=64 | >=64 | >=64 | >=16 | >=16 | >=64 | >=8 | >=4 | >=320 | >=16 | 1 | >=64 | >=16 | 0.5 |
| KP-74 | >=32 | >=32 | >=128 | >=64 | >=64 | >=64 | >=64 | >=16 | >=16 | >=64 | >=8 | >=4 | >=320 | >=16 | 1 | >=64 | >=16 | 0.25 |
| KP-75 | >=32 | >=32 | >=128 | >=64 | 16 | 32 | >=64 | >=16 | >=16 | >=64 | >=8 | >=4 | <=20 | 4 | 0.5 | >=64 | >=16 | 1 |
| KP-76 | >=32 | >=32 | >=128 | >=64 | >=64 | >=64 | >=64 | >=16 | >=16 | >=64 | >=8 | >=4 | >=320 | >=16 | 1 | >=64 | >=16 | 0.5 |
| KP-77 | >=32 | >=32 | >=128 | >=64 | >=64 | >=64 | >=64 | >=16 | >=16 | >=64 | >=8 | >=4 | >=320 | >=16 | 0.5 | 32 | >=16 | 0.5 |
| KP-78 | >=32 | >=32 | >=128 | >=64 | >=64 | >=64 | >=64 | >=16 | >=16 | >=64 | >=8 | >=4 | >=320 | >=16 | 2 | >=64 | >=16 | 1 |
| KP-80 | >=32 | >=32 | >=128 | >=64 | >=64 | >=64 | >=64 | >=16 | >=16 | >=64 | >=8 | >=4 | >=320 | >=16 | 2 | >=64 | >=16 | 1 |
| KP-81 | >=32 | >=32 | >=128 | >=64 | >=64 | >=64 | >=64 | >=16 | >=16 | >=64 | >=8 | >=4 | <=20 | >=16 | 2 | <=2 | <=1 | 1 |
| KP-82 | >=32 | >=32 | >=128 | >=64 | >=64 | >=64 | >=64 | >=16 | >=16 | >=64 | >=8 | >=4 | >=320 | >=16 | 1 | >=64 | >=16 | 4 |
| KP-84 | >=32 | >=32 | >=128 | >=64 | >=64 | >=64 | 32 | >=16 | >=16 | 32 | 0.5 | 0.5 | >=320 | <=1 | 0.5 | <=2 | <=1 | 1 |
| KP-87 | >=32 | >=32 | >=128 | >=64 | >=64 | 32 | >=64 | >=16 | >=16 | >=64 | >=8 | >=4 | >=320 | >=16 | 2 | >=64 | >=16 | 0.5 |
| KP-88 | >=32 | >=32 | >=128 | >=64 | >=64 | <=2 | >=64 | >=16 | >=16 | >=64 | >=8 | >=4 | >=320 | >=16 | 1 | >=64 | >=16 | 0.5 |
| KP-90 | >=32 | >=32 | >=128 | >=64 | >=64 | >=64 | >=64 | >=16 | >=16 | >=64 | >=8 | >=4 | <=20 | >=16 | 2 | 16 | >=16 | 1 |
| KP-91 | >=32 | >=32 | >=128 | >=64 | >=64 | >=64 | >=64 | >=16 | >=16 | >=64 | >=8 | >=4 | >=320 | >=16 | 0.5 | 4 | 4 | 1 |
| KP-92 | >=32 | >=32 | >=128 | >=64 | >=64 | >=64 | >=64 | >=16 | >=16 | >=64 | >=8 | >=4 | >=320 | >=16 | 1 | >=64 | >=16 | 1 |
| KP-94 | >=32 | >=32 | >=128 | >=64 | >=64 | >=64 | >=64 | >=16 | >=16 | >=64 | >=8 | >=4 | >=320 | >=16 | 2 | >=64 | >=16 | 0.5 |
| KP-96 | >=32 | >=32 | >=128 | >=64 | >=64 | >=64 | >=64 | >=16 | >=16 | >=64 | >=8 | >=4 | >=320 | >=16 | 2 | >=64 | >=16 | 1 |
| KP-97 | >=32 | >=32 | >=128 | >=64 | >=64 | >=64 | >=64 | >=16 | >=16 | >=64 | >=8 | >=4 | >=320 | >=16 | 2 | >=64 | >=16 | 64 |
| KP-98 | >=32 | >=32 | >=128 | >=64 | >=64 | >=64 | >=64 | >=16 | >=16 | >=64 | >=8 | >=4 | >=320 | >=16 | 0.5 | >=64 | >=16 | 0.5 |
| KP-99 | >=32 | >=32 | >=128 | >=64 | >=64 | >=64 | >=64 | >=16 | >=16 | >=64 | >=8 | >=4 | >=320 | >=16 | 2 | >=64 | >=16 | 0.5 |
| KP-100 | >=32 | >=32 | >=128 | >=64 | >=64 | >=64 | >=64 | >=16 | >=16 | >=64 | >=8 | >=4 | >=320 | >=16 | 1 | >=64 | >=16 | 1 |
| KP-101 | >=32 | >=32 | >=128 | >=64 | >=64 | >=64 | >=64 | >=16 | >=16 | >=64 | >=8 | >=4 | >=320 | >=16 | 2 | >=64 | >=16 | 0.5 |
| KP-102 | >=32 | >=32 | >=128 | >=64 | >=64 | 32 | >=64 | >=16 | 8 | >=64 | >=8 | >=4 | >=320 | >=16 | 1 | 8 | >=16 | 64 |
| KP-103 | >=32 | >=32 | >=128 | >=64 | >=64 | >=64 | >=64 | >=16 | >=16 | >=64 | >=8 | >=4 | >=320 | >=16 | 0.5 | >=64 | >=16 | 2 |
| **MIC range** | **32** | **32** | **128** | **64** | **16-64** | **2-64** | **16-64** | **8-16** | **8-16** | **4-64** | **0.5-8** | **0.5-4** | **20-320** | **4-16** | **0.5-4** | **4-64** | **1-16** | **0.25-64** |

AMP, ampicillin; ASM, ampicillin/sulbactam; TZP, piperacillin/tazobactam; CEF, cefalotin; CAZ, ceftazidime; FEP, cefepime; SCF, cefoperazone/sulbactam; IPM, imipenem; MEM, meropenem; ATM, aztreonam; CIP, ciprofloxacin; LVX, levofloxacin; AK, amikacin; TOB, tobramycin; SXT, trimethoprim/sulfamethoxazole; TET, tetracycline; TGC, tigecycline; POL, polymyxin B.
